# Reduced Hedonic Valuation of Rewards and Unaffected Cognitive Regulation in Chronic Stress

**DOI:** 10.3389/fnins.2019.00724

**Published:** 2019-07-10

**Authors:** Sónia Ferreira, Carlos Veiga, Pedro Moreira, Ricardo Magalhães, Ana Coelho, Paulo Marques, Carlos Portugal-Nunes, Nuno Sousa, Pedro Morgado

**Affiliations:** ^1^Life and Health Sciences Research Institute (ICVS), School of Medicine, University of Minho, Braga, Portugal; ^2^ICVS/3B’s – PT Government Associate Laboratory, Braga/Guimarães, Portugal; ^3^Clinical Academic Center – Braga, Braga, Portugal

**Keywords:** stress, decision-making, cognition, magnetic resonance imaging, fMRI, reward, human, food

## Abstract

Cognition can influence choices by modulation of decision-making processes. This cognitive regulation is defined as processing information, applying knowledge, and changing preferences to consciously modulate decisions. While cognitive regulation of emotions has been extensively studied in psychiatry, few works have detailed cognitive regulation of decision-making. Stress may influence emotional behavior, cognition, and decision-making. In addition, the brain regions responsible for decision-making are sensitive to stress-induced changes. Thus, we hypothesize that chronic stress may disrupt the ability to regulate choices. Herein, we used a functional magnetic resonance imaging task where fourteen control and fifteen chronically stressed students had to cognitively upregulate or downregulate their craving before placing a bid to obtain food. We found that stressed participants placed lower bids to get the reward and chose less frequently higher bid values for food. Nevertheless, we did not find neural and behavioral differences during cognitive regulation of craving. Our outcomes revealed that chronic stress impacts decision-making after cognitive regulation of craving by reducing the valuation of food rewards but not cognitive modulation itself. Importantly, our results need further validation with larger sample sizes.

## Introduction

Value-based decision-making is the ability to make a choice from competing courses of action/alternatives based on subjective values and possible outcomes attributed to them (Balleine, 2005). This process is carried out whenever a person chooses from different alternatives (e.g., choosing between eating an apple or an orange, or between going out or not). Different interacting systems are responsible for the valuation and action selection processes in the brain (Rangel et al., 2008). First, a valuation system computes the action values. A comparator system needs to evaluate the action values. An accumulator system receives and accumulates the value signals from the comparator system until the signal for one of the actions is sufficiently strong for the choice to be executed (Gold and Shadlen, 2007; Basten et al., 2010).

Values assigned to actions during the valuation process can be influenced by different factors such as the degree of risk or uncertainty of the action (Platt and Huettel, 2008; Rangel et al., 2008). Humans have a natural aversion to risky or uncertain choices and place less value on actions with temporal uncertain rewards or multiple sets of outcomes (Christopoulos et al., 2009; McGuire and Kable, 2012). Individuals often place higher values on immediate rewards rather than on future ones (Rangel et al., 2008). Social competition, cooperation, and concerns for the well-being of others also influence decision-making (Fehr and Camerer, 2007). Cognition can also influence choices through modulation of the decision-making processes. This cognitive regulation process may be defined as processing information, applying knowledge and changing preferences to consciously modulate our decisions. While cognitive regulation of emotional response has been extensively studied (Ochsner et al., 2004; Delgado et al., 2008; Wager et al., 2008), few works have detailed cognitive regulation of decision-making. A functional magnetic resonance imaging (fMRI) study where participants had to modulate their cravings for food showed that cognitive regulation affects decision-making through valuation regulation and behavioral control (Hutcherson et al., 2012). The ventromedial prefrontal cortex (vmPFC) is known to compute the value signal of decisions while the dorsolateral prefrontal cortex (dlPFC) modulates this signal during cognitive regulation tasks (Hare et al., 2009, 2011; Kober et al., 2010).

Cognitive regulation of both emotion and decision-making has a role in the treatment of several conditions (schizophrenia, bipolar disorder, depression, obesity, addiction, obsessive-compulsive disorder, and eating disorders) where emotional processing and decision-making are often impaired (Phillips et al., 2003; Ochsner et al., 2004). On the other hand, mental disorders such as schizophrenia, bipolar disorder, post-traumatic stress disorder, and depression are often associated with prolonged exposure to stress (Arnsten, 2015; Sousa, 2016). Stress impacts emotional processing leading to depressive and anxious behavior associated with alterations in amygdala-ventromedial-prefrontal pathways. Moreover, stress elicits cognitive impairments namely working memory and attentional deficits, poor decision-making (e.g., decreased reward sensitivity or increased influence of immediate rewards), behavioral inflexibility, and learning deficits. These cognitive differences are associated with changes in prefrontal and hippocampal regions (Sandi, 2013; Arnsten, 2015; Chen and Baram, 2016; Sousa, 2016). Additionally, the brain regions implicated in decision-making processes are sensitive to stress-induced changes. In fact, changes in fronto-striatal networks involved in behavioral decisions have been reported in both humans and rodents after chronic stress (Dias-Ferreira et al., 2009; Soares et al., 2012; Morgado et al., 2012, 2015a; Sousa, 2016; Magalhães et al., 2018). Thus, stress seems to influence the quality of decisions (Starcke and Brand, 2012; Morgado et al., 2015b; Bryce and Floresco, 2016; Chen and Baram, 2016) because cognitive control is diminished (Yu, 2016).

Stress has also an impact on appetite and eating behavior (Ans et al., 2018) and is one of the factors for development of eating and obesity-associated conditions (Razzoli et al., 2017). Usually, the production of adrenocorticotropic hormone (ACTH) by the anterior pituitary gland leads to the release of cortisol in the adrenal cortex to stimulate hunger and feeding behavior. High cortisol levels are associated with high insulin concentrations resulting in increased caloric intake or food craving (Adam and Epel, 2007). Stress might boost these pathways leading to an increase in food intake and appetite for high-caloric food, or also reduced reward sensitivity to low-caloric food (Razzoli et al., 2017; Ans et al., 2018; Berg Schmidt et al., 2018; Ferrer-Cascales et al., 2018), in agreement with previous stress decision-making studies demonstrating decreased reward sensitivity or increased influence of immediate rewards (Morgado et al., 2015b). Thus, stress seems to be associated with increased food reward sensitivity due to diminished self-control during food choice associated with decreased functional connectivity between the vmPFC and dlPFC (Neseliler et al., 2017) and increased connectivity between the vmPFC and subcortical regions (amygdala and striatum) (Tryon et al., 2013; Maier et al., 2015).

Herein, we used an fMRI task to clarify the impact of chronic stress on cognitive regulation of decisions. Our task consisted of cognitively upregulating or downregulating craving before placing a bid to obtain food. In addition to brain responses, we analyzed behavioral parameters (food valuation score and reaction time) associated with the task, and blood hormonal changes after the task (insulin, cortisol, and glucose). Regarding the previous findings, we hypothesize that chronic stress may disrupt the ability of individuals to regulate their choices. We expect that cognitive regulation deficits after chronic stress manifest by changes in the prefrontal cortex (vmPFC and dlPFC). Subsequently, these deficits lead to decision-making impairments, namely increased reward sensitivity, underlying brain response alterations in prefrontal and striatal regions. Moreover, we expect that chronic stress participants present augmented levels of insulin, glucose, and cortisol after the stimulation with food pictures due to an increased reward sensitivity to food.

## Materials and Methods

### Subjects

We enrolled in this study medical students from the School of Medicine of University of Minho, Portugal. All students were healthy Caucasians, right-handed, and had a healthy body-mass index. One group was under normal academic activities [control group, *n* = 14; 9 females/5 males; median (range) 23.00 (3.00) years of age; education 17.00 (3.00) years] and the other included subjects on the long period of preparation for the medical licensing exam [chronic psychosocial stress condition; stress group, *n* = 15; 10 females/5 males; 24.00 (3.00) years of age; education 18.00 (0.00) years]. This work was conducted 1 to 3 months before the exam, but students usually start preparing 1 year before the exam. Subjects were eligible if they were at least 18 years old, reported no history of psychiatric or neurological conditions, traumatic brain lesion, or substance abuse, and were not on any psychiatric medication. The groups were matched for gender (chi-squared test *χ*^2^_(1)_ = 0.02, *p* = 0.893) but not for age (Mann–Whitney test *U* = 169.00, *p* = 0.004, effect size *r* = 0.56) and education level (*U* = 210.00, *p* = 2.579 × 10^–8^, *r* = 0.93).

### Ethics Statement

The study was performed in accordance with the Declaration of Helsinki and was approved by Ethics Subcommittee for the Life and Health Sciences of University of Minho, Portugal, and by the Ethics Committee of the Hospital of Braga, Portugal. All subjects were provided with written informed consent following description of the study goals and procedures.

### Sociodemographic and Psychological Scales

Subjects filled a questionnaire to characterize gender, age, educational level, handedness, and ethnic origin. Weight and height were also measured to prevent the inclusion of participants with an unhealthy body mass index. Subjects were assessed with the 10-items Perceived Stress Scale (PSS-10) (Cohen et al., 1983; Morgado et al., 2013), the Beck Anxiety Inventory (BAI) (Beck et al., 1988), and the Beck Depression Inventory (BDI) (Beck et al., 1996). PSS-10 measures the extent to which participants perceived their life as unpredictable, uncontrollable, and overloaded during the previous month. The higher the score, the greater the intensity of perceived stress. BAI measures the severity of an individual’s anxiety during the previous week. Scores lower than 8 indicate minimal anxiety. Scores higher than 7, 15, and 25 indicate mild, moderate, and severe anxiety, respectively. BDI measures the severity of depression and can be used as a screening tool. Scores lower than 14 indicate minimal depression. Higher scores indicate more severe depressive symptoms.

### Blood Sampling and Analysis

Before the fMRI acquisition, samples of venous blood were collected from all participants into a 5 mL potassium ethylenediaminetetraacetic acid tube and a serum tube. We repeated the collection immediately after the fMRI acquisition. Pre-scan blood samples were used to measure cortisol, glucose, insulin, and ACTH serum levels. In post-scan samples, we repeated cortisol, glucose, and insulin serum measurements (ACTH measurement was not repeated due to technical constraints). The collection took place between 2 and 7 pm which assures small variation in cortisol levels during this period (Minkley and Kirchner, 2012). ACTH was measured based on solid-phase, two-site sequential chemiluminescent immunometric assay, and insulin with solid-phase, enzyme-labeled chemiluminescent immunometric assay (IMMULITE 2000, Siemens AG, Germany). Cortisol levels were assessed with competitive immunoassay based on direct chemiluminescent (ADVIA Centaur and Centaur XP, Siemens AG, Germany). Glucose was measured based on the hexokinase-glucose-6-phosphate method (Dimension Vista, Siemens AG, Germany). Standard procedures were applied following the manufacturer instructions.

### Statistical Analysis

Data related with psychological scales, laboratory values, and behavioral parameters were analyzed using IBM SPSS Statistics (version 24.0; IBM Corporation, United States). Kolmogorov–Smirnov and Shapiro–Wilk tests were used to assess for normality in the distribution of data. Comparisons between groups were carried out by parametric *t*-tests or repeated measures ANOVA (*F*-test, with Bonferroni correction for multiple comparisons for *post hoc* tests], or non-parametric Mann-Whitney *U*-tests. Differences were considered statistically significant if *p* < 0.05. Effect sizes were calculated for all statistically significant results.

### fMRI Task

The task was adapted from Hutcherson et al. (2012). Subjects were instructed to fast for at least 4 h before their arrival and to eat a light meal before the fasting period to increase the valuation of food pictures. We also informed that they would remain in the laboratory for 30 min at the end of the experiment to eat the food they obtained during the fMRI task. The task consisted of two parts: a pre-scan rating task that provided us with a measure of the baseline value for food, and an in-scan bidding and regulation task that measured the food value under the influence of regulation.

During the pre-scan rating task, subjects were shown 150 pictures of different snack food items (e.g., cake, chips, and candy) and rated, at their own pace, how much they would like to eat them using a four-point scale (1, “Don’t want it at all”; 4, “Want it a lot”). Our set of pictures was adapted to the Portuguese context of food.

**FIGURE 1:**
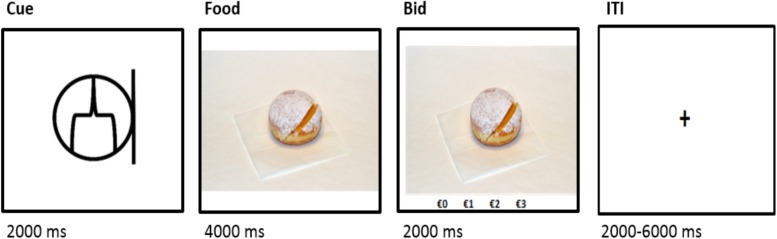
Timing and structure of a trial from the functional magnetic resonance task. ITI, intertrial interval.

Afterward, subjects received instructions for the in-scan bidding and regulation task (Figure 1). The 150 snack food pictures were shown again, separated into three trial conditions: indulge, distance, and natural. Each type of trial appeared 50 times, randomly interspersed over the scanning run. On each trial, before the food appeared, participants saw an abstract black-and-white symbol indicating the trial type (cue, 2 s). On indulge trials, subjects were instructed to try to increase their craving for the snack using any strategy they needed to. During distance trials, the instruction consisted on trying to decrease their craving. On natural trials, they had to allow thoughts and feelings to come naturally. Subjects had 4 s to look at the item and engage in the craving cognitive regulation task (hereinafter referred as cognitive regulation task). After the 4 s, subjects had 2 s to place a bid (0 €, 1 €, 2 €, or 3 €) for the right to eat that food at the end of the experiment. They were asked to treat each trial as if it were the only decision that counted. These bids allowed us to measure values expressed in behavior at the time of choice.

At the end of the experiment, food was auctioned using an adapted version of the Becker-DeGroot-Marschak auction (Becker et al., 1964; Plassmann et al., 2007). We gave 3 € to each subject to spend during the auction over a maximum of three trials. Snacks and snacks prices were randomly selected by drawing a paper from a bag. The bids on those trials during the fMRI task determined whether subjects got to eat that food. Consider *b* the bid made by the subject during the fMRI task. During the auction, a random price *a* was drawn (0 €, 1 €, 2 €, and 3 € were chosen with equal probability). If *b* ≥ *a*, the participant got the item and spend *a*. If *b* < *a*, the subject did not get the item. The rules of the auction ensure the subjects’ best strategy to bid their true value for each food. This was explained and emphasized during the instruction period. For auction effects, omissions resulted in a bid of 0 €.

### fMRI Data Acquisition

Each participant was scanned on a clinical approved 1.5 T Siemens Magnetom Avanto system (Siemens Medical Solutions, Germany) using a 12-channel receive-only head array coil. For the functional acquisition, a T2^*^ weighted echo-planar imaging acquisition was acquired: 38 interleaved axial slices, repetition time 2750 ms, echo time 30 ms, field of view 224 mm × 224 mm, flip angle 90°, in-plane resolution 3.5 mm × 3.5 mm, slice thickness 3.5 mm, and between-slice gap 0.5 mm. To optimize the sensitivity in the orbitofrontal cortex, a tilted acquisition in an oblique orientation of 30° relative to the anterior-posterior commissure line was used. In total, 650 volumes were acquired during the task. The task stimulus was presented using the fully integrated fMRI system IFIS-SA (Invivo Corporation, United States) and the same system was used to record the subject key-press responses. One high-resolution T1-weighted Magnetization-Prepared Rapid Acquisition with Gradient Echo sequence, with 1 mm × 1 mm × 1 mm voxel size, repetition time 2.73 s, echo time 3.48 ms, flip angle 7°, field of view 234 mm × 234 mm, and 176 slices was acquired. This anatomical sequence was used to project the functional maps.

### fMRI Data Preprocessing

The functional scans from each participant were preprocessed using the Statistical Parametric Mapping (SPM) version 12 (Wellcome Department of Imaging Neuroscience, Institute of Neurology, United Kingdom) using MATLAB version R2018a (The MathWorks Inc.,United States). The preprocessing procedures included: slice-timing correction using the first slice as reference; realignment to the mean volume of the acquisition; nonlinear spatial normalization to Montreal Neurological Institute (MNI) standard space and resampling to 2 mm × 2 mm × 2 mm voxel size; spatial smoothing with a 8 mm full-width at half-maximum Gaussian kernel; high pass temporal filtering at 128 s. Participants with more than 3 mm of movement (1 voxel) were excluded (*n* = 0).

### fMRI Data Analysis

For the first-level analysis, one general linear model (GLM) was computed per participant. For this GLM, the regressors of interest included: the type of cognitive regulation trial (1 – distance, 2 – natural, and 3 – indulge) and the corresponding bid (4 – bids after distance trials, 5 – bids after natural trials, and 6 – bids after indulge trials). The bid regressors were parametrically modulated by the bid value (0, 1, 2, and 3 €), the pre-rating score before the task (1 to 4), and the reaction time. Additional regressors included: 7 – the cue; 8 – the interstimulus interval; 9 – the omission bids; 10 – 16 the motion parameters estimated during the realignment step. The onset and duration of the regressors were defined accordingly to the stimulus represented in Figure 1 with a boxcar function and the regressors were convolved with the canonical hemodynamic response function.

At the group level (second-level analysis), a random-effects analysis was performed using four different mixed-design ANOVA models: (1) represented the cognitive regulation during the task (enabled comparisons in average activation for each regulation trial between and within groups); (2) concerned the bidding/valuation during the task modulated by the bid value; (3) concerned the bidding/valuation during the task modulated by the pre-rating score; (4) concerned the bidding/valuation during the task modulated by the reaction time. Models (2) – (4) were used to test if food valuation was different between groups after distinct regulation trials. For all models, the group (stress vs. control) was introduced as the between-subject factor and each trial during cognitive regulation (distance vs. natural vs. indulge) as the within-subject factor. Age and education were used as covariates for all models. All models were implemented with the GLMFlex toolbox^1^ which uses partitioned error terms for within-group and between-group comparisons, enabling the estimation of all the effects of interest with a single model.

Results were considered statistically significant after correcting for multiple comparisons using cluster correction (minimum cluster size of 90 voxels). The minimum cluster size was determined with 3DClustSim (AFNI version 17.0.13; National Institute of Mental Health)^2^. This program determines a minimum cluster size with Monte Carlo Simulation to achieve a corrected significance of *p* < 0.05 with an initial voxel-wise threshold of *p* < 0.001. The Automated Anatomical Labeling plugin for SPM was used to classify the brain regions.

## Results

### Psychological Assessment

The stress group revealed higher levels of perceived stress (mean ± standard deviation 15.07 ± 5.23) than the control group (8.64 ± 5.27) as assessed by PSS-10 [*t*_(27)_ = 3.30, *p =* 0.003, effect size *d* = 1.27]. No statistically significant differences were found for BAI (*U* = 117.50, *p* = 0.591) and BDI (*U* = 134.00, *p* = 0.217) between groups.

### Blood Sampling

The ACTH levels before the fMRI session were similar between the two groups (*U* = 81.50, *p* = 0.310).

Cortisol serum levels were not statistically significantly different between groups [group *F*_(1,27)_ = 0.45*, p* = 0.509] nor within group before and after the fMRI session [group × time *F*_(1,27)_ = 1.00 × 10^–3^*, p* = 0.971]. However, cortisol levels decreased in both groups after the task [time *F*_(1,27)_ = 10.08*, p* = 0.004, effect size *χ*^2^ = 0.27].

Glucose serum levels were not statistically significantly different between groups [group *F*_(1,27)_ = 0.40, *p* = 0.531] and the pre and post-measurement were similar within groups [group × time *F*_(1,27)_ = 0.18, *p* = 0.672]. However, glucose levels decreased in both groups after the task [time *F*_(1,27)_ = 8.44*, p* = 0.007, *χ*^2^ = 0.24].

Insulin serum levels were not statistically significantly different between groups [group *F*_(1,27)_ = 0.42, *p* = 0.522] and the pre and post-measurement were similar within groups [group × time *F*_(1,27)_ = 3.68, *p* = 0.066]. However, insulin levels decreased in both groups after the task [time *F*_(1,27)_ = 9.21, *p* = 0.005, *χ*^2^ = 0.25].

### Behavioral Analysis

Given the differences in age and education between groups, we used these variables as covariates when analyzing behavioral parameters.

**FIGURE 2:**
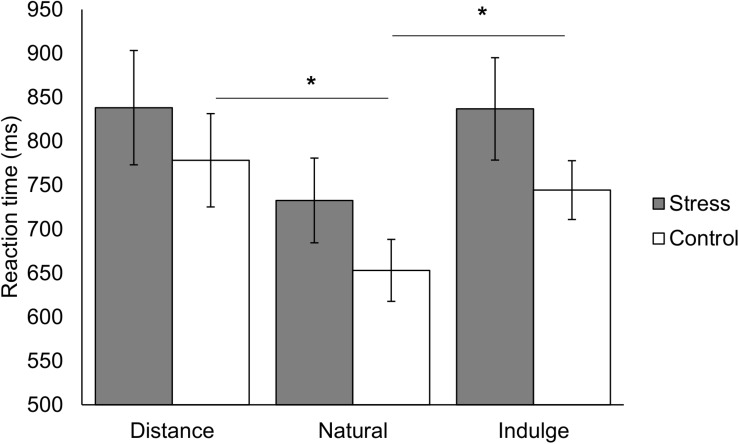
Representation of the reaction time for the different regulation conditions (natural, distance, and indulge) for the stress and control group. The reaction time for natural trials was shorter than for distance [*t*_(13)_ = 4.82, *p =* 0.001, *d* = 2.67] and indulge trials [*t*_(13)_ = 3.07, *p =* 0.027, *d* = 1.70] in the control group but no statistically significant differences occurred in the stress group. The black star represents statistically significant differences. The main bars represent the mean values and the error bars represent the standard error.

We analyzed the reaction time between and within groups during the different regulation trials (distance, natural, and indulge). We found an interaction effect between the group and the reaction time across the different regulation conditions [group × condition *F*_(2,50)_ = 4.00, *p* = 0.024, *χ*^2^ = 0.14; Table 1 represents the results for all between and within group factors and covariate effects]. *Post hoc* tests with repeated measures ANOVA demonstrated statistically significant reaction time differences within the control group [*F*_(1.42,18.43)_ = 7.06, *p* = 0.010, *χ*^2^ = 0.35, Greenhouse-Geisser correction for non-sphericity] and within the stress group [*F*_(1.34,18.82)_ = 4.72, *p* = 0.033, *χ*^2^ = 0.25, Greenhouse-Geisser correction for non-sphericity]. Paired *t*-tests with Bonferroni correction showed that the reaction time for natural trials was shorter than for distance [*t*_(13)_ = 4.82, *p =* 0.001, *d* = 2.67] and indulge trials [*t*_(13)_ = 3.07, *p =* 0.027, *d* = 1.70], and distance and indulge trials presented similar reaction times [*t*_(13)_ = 1.00, *p =* 1.000] in the control group. However, we did not find significant statistical differences in the stress group during post-hoc analysis [1.00 ≤ *t*_(14)_ ≤ 2.36, 0.099 ≤ *p* ≤ 1.000] (Figure 2).

**TABLE 1:** Results for statistical tests on behavioral variables associated with the functional magnetic resonance imaging task: reaction time, valuation score, and response frequency.

**Statistical effect**	**Test value**	***P*-value**	**Effect size *χ*^2^**
**Reaction time**			
Condition (distance, natural, and indulge)	*F*_(2,50)_ = 3.00	0.059	
Group × condition	*F*_(2,50)_ = 4.00	0.024	0.14^*^
Group	*F*_(1,25)_ = 0.02	0.886	
Age	*F*_(1,25)_ = 1.36 × 10^–4^	0.991	
Education	*F*_(1,25)_ = 0.40	0.535	
**Valuation score**			
*Pre-rating score*			
Condition (distance, natural, and indulge)	*F*_(2, 50)_ = 1.21	0.308	
Group × condition	*F*_(2, 50)_ = 0.85	0.433	
Group	*F*_(1, 25)_ = 0.32	0.574	
Age	*F*_(1, 25)_ = 0.03	0.873	
Education	*F*_(1, 25)_ = 0.12	0.728	
*Bid value*			
Condition (distance, natural, and indulge)	*F*_(1.32, 33.03)_ = 0.57	0.502	
Group × condition	*F*_(1.32, 33.03)_ = 1.87	0.180	
Group	*F*_(1, 25)_ = 6.91	0.014	0.22^*^
Age	*F*_(1, 25)_ = 0.11	0.746	
Education	*F*_(1, 25)_ = 0.56	0.462	
**Response frequency**			
Condition (distance, natural, and indulge)	*F*_(1.16, 29.0)_ = 0.02	0.912^a^	
Group × condition	*F*_(1.16, 29.0)_ = 0.03	0.895^a^	
Valuation (0, 1, 2, and 3 €)	*F*_(2.13, 53.15)_ = 1.30	0.283^a^	
Group × valuation	*F*_(2.13, 53.15)_ = 3.89	0.024^a^	0.13^*^
Condition × valuation	*F*_(3.44, 86.11)_ = 0.76	0.536^a^	
Group × condition × valuation	*F*_(3.44, 86.11)_ = 1.45	0.231^a^	
Group	*F*_(1, 25)_ = 0.27	0.605	
Age	*F*_(1, 25)_ = 0.20	0.655	
Education	*F*_(1, 25)_ = 0.11	0.747	

^*^Statistical significance; ^*a*^Greenhouse-Geisser correction for non-sphericity.

**FIGURE 3:**
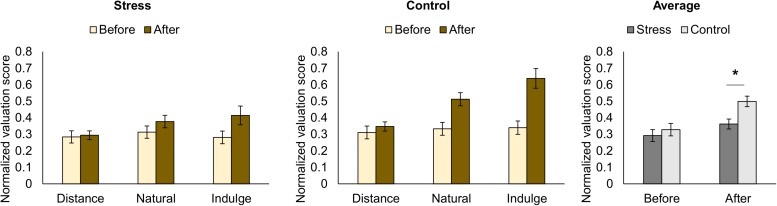
Representation of the normalized rating scores for food pictures for each trial condition (natural, distance, and indulge) before performing the functional magnetic resonance task (before cognitive regulation) and during the functional magnetic resonance task (after cognitive regulation) for the stress and control groups (the normalized scores represent the ratio between given score and maximum score). Before cognitive regulation, we did not find statistically significant differences between groups or within group in terms of food valuation across the conditions. After cognitive regulation, the stress group had lower average valuation scores in comparison to the control group [group *F*_(1,25)_ = 6.91, *p* = 0.014, *χ*^2^ = 0.22]. The black star represents statistically significant differences. The main bars represent the mean values and the error bars represent the standard error.

Taking into account that different instructions were given during the pre-rating (how much the participants want the food) and the bidding (how much the participants want to pay for the food), we separately analyzed differences between groups in the valuation score across the regulation conditions (distance, natural, and indulge) for pre and post-regulation scores (Table 1 represents the results for all between and within group factors and covariate effects). During pre-rating, we did not find statistically significant differences between groups or within group in terms of food valuation across the conditions. Moreover, the valuation score varied similarly among the conditions for both groups. However, during bidding, we found differences between groups [group *F*_(1,25)_ = 6.91, *p* = 0.014, *χ*^2^ = 0.22] but not within group in terms of food valuation across the conditions. Moreover, the valuation score varied similarly among the conditions for both groups. The stress group had lower valuation scores (1.06 ± 0.36 €) during bidding in comparison to the control group (1.50 ± 0.36 €) (Figure 3).

**FIGURE 4:**
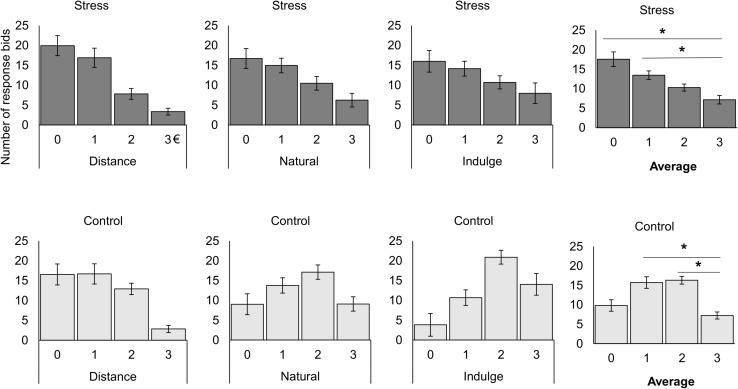
Representation of the number of responses for the stress and control groups during bidding for each cognitive regulation trial and for the average of all trials. On average, the stress participants bided more often 0 [*t*_(14)_ = 3.93, *p =* 0.009, *d* = 1.78] and 1 € [*t*_(14)_ = 3.38, *p =* 0.027, *d* = 1.81] than 3 €, while control subjects bided more times 1 [*t*_(13)_ = 3.94, *p =* 0.010, *d* = 2.18] and 2 € [*t*_(13)_ = 5.91, *p =* 3.090 × 10^–4^, *d* = 3.28] than 3 €. The black star represents statistically significant differences. The main bars represent the mean values and the error bars represent the standard error.

Moreover, we also studied differences between groups in the number of responses for each bidding value after each cognitive regulation trial inside the scanner. We found a significant interaction effect between the group and the bid value [group × valuation *F*_(2.13,53.15)_ = 3.89, *p* = 0.024, *χ*^2^ = 0.13, Greenhouse-Geisser correction for non-sphericity; Table 1 represents the results for all between and within group factors and covariate effects]. *Post hoc* tests with repeated measures ANOVA demonstrated that the control [*F*_(3,39)_ = 9.61, *p* = 7.000 × 10^–5^, *χ*^2^ = 0.42] and the stress group [*F*_(2.03,28.43)_ = 9.04, *p* = 0.001, *χ*^2^ = 0.39, Greenhouse-Geisser correction for non-sphericity] had a different number of responses across the bid values. Paired *t*-tests with Bonferroni correction demonstrated that on average the stress participants bided more often 0 [*t*_(14)_ = 3.93, *p =* 0.009, *d* = 1.78] and 1 € [*t*_(14)_ = 3.38, *p =* 0.027, *d* = 1.81] than 3 €, while control subjects bided more times 1 [*t*_(13)_ = 3.94, *p =* 0.010, *d* = 2.18] and 2 € [*t*_(13)_ = 5.91, *p =* 3.090 × 10^–4^, *d* = 3.28] than 3 € (Figure 4).

### Neuroimaging Results

**FIGURE 5:**
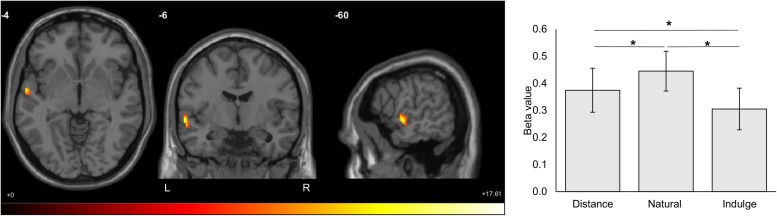
Statistically significant brain regions resulting from the main effect of the cognitive regulation condition (natural, indulge, and distance) for both groups (cluster correction for multiple comparisons, minimum voxel size of 90, *p* < 0.001, *F*_(2,54)_ value between 7.87 and 13.97 represented by the colored bar). The distance and indulge trials elicited lower activity than natural trials, and the distance trials lead to higher responses than indulge trials in these regions (distance vs. natural *t*_(28)_ = 2.97, *p =* 0.018, *d* = 1.12; distance vs. indulge *t*_(28)_ = 2.68, *p =* 0.036, *d* = 1.01; natural vs. indulge *t*_(28)_ = 4.97, *p =* 9 × 10^– 5^, *d* = 1.88). These regions include the superior (Brodmann area 22) and middle temporal gyrus (Brodmann area 21), the rolandic operculum, and the precentral gyrus (Brodmann area 6). The numbers above the slices represent the Montreal Neurological Institute peak voxel coordinates. The black star represents statistically significant differences. The main bars represent the mean values and the error bars represent the standard error. L, left; R, Right.

We tested for differences in blood-oxygen-level-dependent responses between stress and control groups during each cognitive regulation period/trial (natural, indulge, and distance) – model (1). No statistically significant brain regions were identified for overall differences between groups (main effect of group). When looking at the interaction effect between trial condition and group, there were also no statistically significant effects. Nonetheless, we found a main effect of the cognitive regulation condition in the left hemisphere in the superior (Brodmann area 22) and middle temporal gyrus (Brodmann area 21), the rolandic operculum, and the precentral gyrus (Brodmann area 6) [7.87 ≤ *F*_(2,54)_ ≤ 13.97, *p* ≤ 0.001, 99 voxels, Montreal Neurological Institute peak voxel coordinates -60 -6 -4). *Post hoc* paired *t*-tests with Bonferroni correction demonstrated that the distance and indulge trials elicited lower activity than natural trials and that distance trials lead to higher responses than indulge trials in these regions [distance vs. natural *t*_(28)_ = 2.97, *p =* 0.018, *d* = 1.12; distance vs. indulge *t*_(28)_ = 2.68, *p =* 0.036, *d* = 1.01; natural vs. indulge *t*_(28)_ = 4.97, *p =* 9 × 10^–5^, *d* = 1.88] (Figure 5).

With the models (2) – (4), we tested if food valuation/bidding behavior was associated with different brain activation between groups after each regulation condition, with parametric modulation by bid value (model 2), pre-rating score (model 3), and reaction time (model 4). No statistically significant regions were identified for overall differences between groups during bidding (main effect of group) for the models (2) – (4). Additionally, no statistically significant active regions were found for interaction effects of group and the bids after each category of cognitive regulation (group × cognitive regulation condition), and the main effect of the condition was also not statistically significant for the models (2) – (4).

### Task Validity

Given that our fMRI task was adapted from Hutcherson et al. (2012), here we compared our main results with these authors’ significant findings to study the task validity. Since we observed behavioral differences between the control and stress groups, we assessed the validity of the task only with the control group.

As observed by Hutcherson et al. (2012), we also saw that the control group took longer while bidding after distance [*t*_(13)_ = 4.82, *p =* 0.001, *d* = 2.67, with Bonferroni correction] and indulge trials [*t*_(13)_ = 3.07, *p =* 0.027, *d* = 1.70, with Bonferroni correction] than natural trials [group × condition *F*_(1.42,18.43)_ = 7.06, *p* = 0.010, *χ*^2^ = 0.35, Greenhouse-Geisser correction for non-sphericity] (Figure 2). Moreover, the distance trials were also associated with the longest reaction time (778.30 ± 198.70 ms), followed by indulge (744.38 ± 125.21 ms), and natural (652.98 ± 131.97 ms) trials. These reaction times values are consistent with the previous study.

Concerning the bid value, similarly to Hutcherson et al. (2012), we observed a main effect of the cognitive regulation condition (distance, natural, and indulge) in controls [*F*_(1.23,16.05)_ = 16.88, *p* = 4.650 × 10^–4^, *χ*^2^ = 0.56, Greenhouse-Geisser correction for non-sphericity]. The control participants bided higher on indulge [1.74 ± 0.43 €; *t*_(13)_ = 2.89, *p =* 0.038, *d* = 1.60, with Bonferroni correction] and lower on distance [1.04 ± 0.28 €; *t*_(13)_ = 4.22, *p =* 0.003, *d* = 2.34, with Bonferroni correction] compared to natural trials (1.50 ± 0.23 €). Bids after distance and indulge trials were also statistically significantly different [*t*_(13)_ = 4.22, *p =* 0.003, *d* = 2.34, with Bonferroni correction] (Figure 3). The bid values in our study (0, 1, 2, and 3 €) were distinct from the original study ($ 0.0, 0.5, 1.0, 1.5, 2.0, and 2.5), thus we could not compare the average bid values after each condition trial.

For neuroimaging data, we computed the contrasts among the cognitive regulation trials in the control group to compare our results with the original study: Distance > Natural, Natural > Distance, Indulge > Natural, Natural > Indulge, Distance > Indulge, and Indulge > Distance. We applied cluster correction for multiple comparisons (90 voxels as described in the section “Materials and Methods”). We found statistically significant results only for the contrasts Distance > Natural and Indulge > Natural. These results are in agreement with Hutcherson et al. (2012)’s findings if the same minimum cluster size is considered. For the contrast Distance > Natural, similarly to the original work, we also found statistically significant activation in temporal and posterior parietal regions (Supplementary Table S1). However, results did not show statistically significant activity in medial and ventrolateral prefrontal regions. For the contrast Indulge > Natural, we found statistically significant responses in the anterior cingulate cortex, the ventral, medial, and superior prefrontal cortex, temporal and parietal regions, and the supplementary motor area (Supplementary Table S1). Thus, our results are concordant with these authors’ previous findings.

## Discussion

We studied how chronic stress influences decision-making on food valuation after cognitive regulation (increasing/indulge or decreasing/distance food craving) in medical students. Behavioral, biochemical and neuroimaging analysis were performed to address this question. We found that stressed participants present decreased food valuation scores. This result was reinforced by a higher number of responses for the lowest bid values for food in the stress group. The biochemical analysis (serum levels of insulin, cortisol, and glucose) did not show statistically significant differences between the control and stress group. The neuroimaging results did not demonstrate statistically significant differently activated brain regions between the stressed and control participants during cognitive regulation of craving and decision-making/bidding.

Although the acute stress response is generally beneficial, i.e., promotes adaptation to stressful stimulus, prolonged activation of the stress response produces deleterious effects on the body and brain, affecting cognitive processes such as decision-making (Mcewen, 2004; McEwen and Gianaros, 2011; Sousa, 2016). One of the main findings of the present study is that stressed individuals presented lower scores during food valuation, in contrast with our initial hypothesis. This may translate a blunted hedonic capacity or reward sensitivity (Berenbaum and Connelly, 1993; Porcelli et al., 2012; Bryce and Floresco, 2016; Porcelli and Delgado, 2017; Uy and Galván, 2017), as anhedonia has been associated with higher perceived stress scale scores (Pizzagalli et al., 2007) and stress causes changes in regions related to hedonic/rewarding behavior such as the amygdala, orbitofrontal cortex, vmPFC, and ventral and dorsal striatum (Gorwood, 2008; Porcelli et al., 2012; Bessa et al., 2013; Stark et al., 2015). Moreover, the distribution of the number of responses was higher for lower bids in the stress group, i.e., stressed subjects seemed less prone to place high bids for food. A previous work including a food-related task discovered decreased reward sensitivity associated with alterations in the putamen activity after acute stress induction (Born et al., 2010). Another report pointed out that acute stress does not potentiate craving after stimulation with food pictures (Stojek et al., 2015). Moreover, animal research indicates that acute stress reduces the motivation to work for food rewards (Bryce and Floresco, 2016). Other studies have also shown that acute and chronic stress mitigate brain responses to food stimuli in reward pathways (Wierenga et al., 2018). These results support the idea that stress participants have a reduced valuation attributed to food rewards. However, other studies have shown increased sensitivity to high-caloric food rewards in stressed individuals (Razzoli et al., 2017; Ans et al., 2018; Berg Schmidt et al., 2018; Ferrer-Cascales et al., 2018). Reward processing might be different when participants are stimulated with food pictures or real food. Moreover, the inclusion of chronic or acute stress models might also account for different results regarding reward sensitivity (Porcelli and Delgado, 2017). However, our results need confirmation with larger sample sizes.

While bidding, the stressed subjects did not present differential brain activity when compared to control subjects, despite the behavioral differences in the valuation score. We were expecting that poor cognitive self-control reflected in reduced prefrontal activation (Hare et al., 2009, 2011; Kober et al., 2010; Hutcherson et al., 2012) would lead to higher responses in striatal and amygdalar regions associated with increased reward sensitivity (Louis et al., 2009; Tryon et al., 2013; Maier et al., 2015; Neseliler et al., 2017). Other studies have found controversial results demonstrating that reduced striatal activity was associated with high levels of stress and increased food craving (Hommer et al., 2013). However, we did not observe cognitive differences between the groups and reward sensitivity was decreased. Our sample size may have limited the statistical power of this analysis. Thus, further research should be conducted to understand the neural correlates of decision-making after cognitive regulation since our results are not conclusive.

Our neuroimaging results did not show brain activity differences between groups during cognitive regulation. According to previous studies, the vmPFC and dlPFC are regions responsible for cognitive regulation in this decision-making context (Hare et al., 2009, 2011; Kober et al., 2010; Hutcherson et al., 2012). Thus, the absence of changes in these regions between groups in our work might indicate that the processes for cognitive regulation of food craving are not affected by chronic stress, or that our specific model of chronic stress might not lead to changes in cognitive modulation of craving. However, previous works revealed that cognitive control is diminished under stress, leading to emotional and habitual-biased decision-making (Yu, 2016), and increased reward sensitivity for food (Tryon et al., 2013; Maier et al., 2015; Neseliler et al., 2017). Our moderate sample size might have hindered putative differences between groups. Nonetheless, as shown in Figures 2, 3, stressed participants were able to modulate their responses, demonstrating an effective cognitive regulation, although the average food bidding score was lower than in controls. Both groups were capable of effectively using cognitive regulation to change the value placed on food during regulated trials. Moreover, both groups took longer times during bidding after regulated trials. Indeed, we found that activity in the superior and middle temporal gyrus, rolandic operculum, and precentral gyrus was differently modulated by trials with cognitive regulation of craving versus non-regulated trials in both groups. Previous authors provided evidence for a functional connection between the vmPFC and the precentral gyrus during food-related decisions, and for the correlation between food ratings and the response in the middle temporal gyrus (Kober et al., 2010; Hare et al., 2011; Hutcherson et al., 2012). Moreover, the temporal gyrus is also involved in food imagery (Hommer et al., 2013). Thus, the regulatory success does not seem to be affected by stress. During cognitive control tasks, attentional narrowing might occur after stimulation with negative pictures with threat and sadness-related content (van Steenbergen et al., 2011; Melcher et al., 2012; Papazacharias et al., 2015). Thus, the negative emotional state in the stress group (e.g., fear of falling the final exam) might have led to higher attentional focus during cognitive regulation that might compensate cognitive deficits associated with chronic stress. Nonetheless, our results need further validation with larger sample sizes to rule out a putative effect of chronic stress in cognitive regulation of craving.

Insulin, cortisol, and glucose levels are expected to decrease after fasting (Kirschbaum et al., 1997; Adam and Epel, 2007; Figlewicz, 2015; Tiedemann et al., 2017). However, peripheral concentrations of cortisol rise after stimulation with food images due to appetite enhancement, while insulin and glucose levels seem to be unaffected (Schmid et al., 2005; Schüssler et al., 2012; Kroemer et al., 2013). In our study, both groups presented a decreased in insulin, glucose, and cortisol levels after the fMRI task. Thus, the effects of fasting might have potentially surpassed the effects of stimulation with food pictures (Brede et al., 2017). Nonetheless, this hypothesis needs further testing. We were expecting increased craving in the stress group after a deficient cognitive regulation and increased reward sensitivity to food (Tryon et al., 2013; Maier et al., 2015; Neseliler et al., 2017). However, our results agree with the fact that we found reduced valuation of rewards in the absence of cognitive regulation alterations in the stress group, suggesting that overall craving was reduced. For controls, the instructions to differently modulate craving might have led to balanced changes in blood parameters after stimulation with food pictures. Thus, our results might derive from fasting since they occurred in both groups.

Importantly, our results are limited by the sample size. These results need to be replicated with larger samples to avoid false negative and positive conclusions. Moreover, the results might have been influenced by the unbalanced proportion of females and males per group, given that gender differences were found in decision-making under stress (Yu, 2016; Wemm and Wulfert, 2017). However, we focused on group differences and groups were matched for gender ratio.

Our results show that the capacity to perform cognitive regulation of craving is not impaired after prolonged stress. However, chronic stress reduces the value attributed to food rewards after craving modulation. Importantly, our conclusions are limited by the small sample size and need further validation with larger samples. These findings are relevant to guide subsequent studies on cognitive regulation of food-related decision-making for eating and obesity-associated disorders. Cognitive control techniques might be used to tackle decision-making impairments in these conditions (Louis et al., 2009; May et al., 2012).

## Data Availability

The datasets generated for this study are available on request to the corresponding author.

## Ethics Statement

This study was carried out in accordance with the Declaration of Helsinki. All subjects gave written informed consent. The protocol was approved by the Ethics Subcommittee for the Life and Health Sciences of University of Minho, Portugal, and by the Ethics Committee of the Hospital of Braga, Portugal.

## Author Contributions

SF, CV, PM, RM, AC, PMore, and CP-N acquired, analyzed, and interpreted the data and wrote the manuscript. NS and PMorg supervised the work, interpreted the data, and wrote and reviewed the manuscript. All authors participated in the design of the experiments and approved the manuscript.

## Conflict of Interest Statement

The authors declare that the research was conducted in the absence of any commercial or financial relationships that could be construed as a potential conflict of interest.

## Footnotes

1 http://nmr.mgh.harvard.edu/harvardagingbrain/People/AaronSchultz/GLM_Flex.html

2 https://afni.nimh.nih.gov/

## Abbreviations

ACTH: adrenocorticotropic hormone
BAI: Beck Anxiety Inventory
BDI: Beck Depression Inventory
dlPFC: dorsolateral prefrontal cortex
fMRI: functional magnetic resonance imaging
GLM: general linear model
MNI: Montreal Neurological Institute
PSS-10: 10-items Perceived Stress Scale
vmPFC: ventromedial prefrontal cortex.
