# Corrigendum: Reduced Hedonic Valuation of Rewards and Unaffected Cognitive Regulation in Chronic Stress

**DOI:** 10.3389/fnins.2019.01252

**Published:** 2019-11-22

**Authors:** Sónia Ferreira, Carlos Veiga, Pedro Moreira, Ricardo Magalhães, Ana Coelho, Paulo Marques, Carlos Portugal-Nunes, Nuno Sousa, Pedro Morgado

**Affiliations:** ^1^Life and Health Sciences Research Institute (ICVS), School of Medicine, University of Minho, Braga, Portugal; ^2^ICVS/3B's – PT Government Associate Laboratory, Braga/Guimarães, Portugal; ^3^Clinical Academic Center – Braga, Braga, Portugal

**Keywords:** stress, decision-making, cognition, magnetic resonance imaging, fMRI, reward, human, food

In the original article, there was an error. The psychometric scale used to measure the depression scores was the “Beck Depression Inventory” and not the “Beck Depression Inventory II.”

A correction has been made to the **Materials and Methods**, subsection **Sociodemographic and Psychological Scales**:

“Subjects filled a questionnaire to characterize gender, age, educational level, handedness, and ethnic origin. Weight and height were also measured to prevent the inclusion of participants with an unhealthy body mass index. Subjects were assessed with the 10-items Perceived Stress Scale (PSS-10) (Cohen et al., 1983; Morgado et al., 2013), the Beck Anxiety Inventory (BAI) (Beck et al., 1988), and the Beck Depression Inventory (BDI) (Beck et al., 1996). PSS-10 measures the extent to which participants perceived their life as unpredictable, uncontrollable, and overloaded during the previous month. The higher the score, the greater the intensity of perceived stress. BAI measures the severity of an individual's anxiety during the previous week. Scores lower than 8 indicate minimal anxiety. Scores higher than 7, 15, and 25 indicate mild, moderate, and severe anxiety, respectively. BDI measures the severity of depression and can be used as a screening tool. Scores lower than 14 indicate minimal depression. Higher scores indicate more severe depressive symptoms.”

A correction has also been made to **Results**, subsection **Psychological Assessment**:

“The stress group revealed higher levels of perceived stress (mean ± standard deviation 15.07 ± 5.23) than the control group (8.64 ± 5.27) as assessed by PSS-10 [*t*_(27)_ = 3.30, *p* = 0.003, effect size *d* = 1.27]. No statistically significant differences were found for BAI (*U* = 117.50, *p* = 0.591) and BDI (*U* = 134.00, *p* = 0.217) between groups.”

Lastly, a correction has been made to the **Abbreviations** section:

“ACTH, adrenocorticotropic hormone; BAI, Beck Anxiety Inventory; BDI, Beck Depression Inventory; dlPFC, dorsolateral prefrontal cortex; fMRI, functional magnetic resonance imaging; GLM, general linear model; MNI, Montreal Neurological Institute; PSS-10, 10-items Perceived Stress Scale; vmPFC, ventromedial prefrontal cortex.”

The authors apologize for this error and state that this does not change the scientific conclusions of the article in any way. The original article has been updated.
