# Supplementary material for: Reduced Hedonic Valuation of Rewards and Unaffected Cognitive Regulation in Chronic Stress
Source: Front Neurosci. 2019 Jul 10;13:724. doi: 10.3389/fnins.2019.00724 (PMC6636668; doi:10.3389/fnins.2019.00724)
Supplement: Supplementary file 1 [file Data_Sheet_1.PDF]

Table S1 Differential brain activity during cognitive regulation trials (distance, natural, and indulge) within the control group ($p < 0.001$; cluster correction with a minimum of 90 voxels).

Brain regions	Cluster size (voxels)	MNI peak voxel coordinates	Peak voxel intensity
**Distance > Natural**			
L Calcarine; Posterior cingulate (BA 30); Lingual gyrus; Cuneus; Temporal lobe.	90	-18 -64 6	$t_{(54)} = 4.37$
**Natural > Distance**			
No statistically significant regions.			
**Indulge > Natural**			
L Fusiform gyrus (BA 37); Lingual gyrus; Parahippocampal gyrus (BA 36); Cerebellum (anterior and posterior lobe).	448	-28 -42 -22	$t_{(54)} = 4.89$
L Superior (BA 22), middle (BA 21), inferior (BA 20), and transverse (BA 41) temporal gyrus; Superior and middle temporal pole (BA 38); Precentral (BA 4 and 6) and postcentral gyrus; Inferior (BA 44 and 47) and middle frontal gyrus (BA 9); Insula/claustrum (BA13); Rolandic operculum; Inferior orbitofrontal; Inferior frontal operculum; Putamen; Fusiform gyrus.	1944	-50 10 -4	$t_{(54)} = 6.14$
L Parahippocampal gyrus (BA 28 and 34); Amygdala; Superior temporal pole; Subcallosal gyrus; Inferior orbitofrontal.	132	-20 4 -20	$t_{(54)} = 4.53$
R Parahippocampal gyrus (BA 28 and 34); Amygdala; Inferior frontal gyrus (BA 47); Subcallosal gyrus; Insula (BA 13); Superior temporal pole; Gyrus rectus; Inferior orbitofrontal.	153	22 8 -20	$t_{(54)} = 4.48$
R, L Anterior, posterior, and middle cingulate; Lingual gyrus; Precuneus; Cuneus; Cerebellum;<br>R Medial and superior frontal gyrus; Supplementary motor area; Calcarine; Parahippocampal gyrus;<br>L Paracentral lobule; Hippocampus.	2512	-8 -40 6	$t_{(54)} = 5.47$
R Superior (BA 22), middle (BA 21), and transverse temporal gyrus; Insula (BA 13); Superior temporal pole (BA 38); Rolandic operculum; Precentral gyrus.	303	64 -8 -2	$t_{(54)} = 5.25$
R Superior (BA 22), middle (BA 21), and transverse temporal gyrus (BA 42).	278	62 -28 4	$t_{(54)} = 4.84$
R, L Medial (BA 9) and superior frontal gyrus; Anterior (BA 24 and 32) and middle cingulate.	408	16 42 48	$t_{(54)} = 5.23$
R Precentral (BA 4) and postcentral gyrus (BA 3); Superior, inferior, and middle frontal gyrus (BA 9).	550	62 -4 36	$t_{(54)} = 4.68$
R, L middle cingulate gyrus (BA 32); Supplementary motor area (BA 8); Medial and superior frontal gyrus.	96	4 20 44	$t_{(54)} = 3.96$
L Precuneus; Parietal lobe (BA 5 and 7); Paracentral lobule.	102	-4 -54 50	$t_{(54)} = 4.17$
L Precentral (BA 4) and postcentral gyrus (BA 1, 2, and 3); Superior parietal lobe (BA 5).	97	-30 -40 66	$t_{(54)} = 4.39$
R Precentral (BA 4) and postcentral gyrus (BA 2 and 3); Parietal lobe; Paracentral lobule; Medial frontal gyrus.	97	24 -34 72	$t_{(54)} = 4.14$
**Natural > Indulge**			
No statistically significant regions.			
**Distance > Indulge**			
No statistically significant regions.			
**Indulge > Distance**			
No statistically significant regions.			

MNI = Montreal Neurologic Institute; L = Left; R = Right; BA = Brodmann Area.
